# The use of ozone in oral health care: a narrative review of current evidence and clinical applications

**DOI:** 10.3389/froh.2026.1846309

**Published:** 2026-06-18

**Authors:** Catherine Jauregui, Gary Chike, Shawyun Khoshneviszadeh, Daniya Killedar

**Affiliations:** Dental College of Georgia, Augusta University, Augusta, GA, United States

**Keywords:** antimicrobial, gaseous ozone, ozonated oils, ozonated water, ozone therapy

## Abstract

**Background:**

Ozone therapy, a clinical application of various ozone forms that is used for its antiseptic techniques, has sparked interest in recent years over its unique antimicrobial and anti-inflammatory properties in the body. Moreover, its ability to work as an antiseptic while avoiding antimicrobial resistance has sparked interest in the dental field as no other antiseptic agent is able to do so. As a triatomic molecule of oxygen (O₃), ozone functions through oxidative mechanisms that disrupt microbial cell walls, inhibit biofilm formation, and modulate host immune responses without contributing to antimicrobial resistance. Types of study reviewed: Peer-reviewed articles published in English, including systematic reviews, meta-analyses, observational studies, narrative reviews, case series, and case reports were included in the article.

**Results:**

This narrative review synthesizes current evidence on the mechanisms of ozone as well as the therapeutic efficacy of ozone in clinical applications across multiple dental specialties. Three main delivery modalities (gaseous ozone, ozonated water, and ozonated oils) demonstrate diverse clinical benefits and are discussed in the review.

**Practical implications:**

Comparative studies indicate that for some dental applications ozone achieves antimicrobial efficacy comparable to conventional agents such as chlorhexidine gluconate (CHX) while exhibiting reduced cytotoxicity.

## Introduction

Ozone (O₃), a triatomic allotrope of oxygen, is well known for its ability to filter ultraviolet radiation in the atmosphere. However, its oxidative capabilities have grown attention in the healthcare field, particularly dentistry, in recent years. As the demand for effective antimicrobial agents continues to grow in dentistry, it is necessary to compare ozone with chemical agents currently in use. Chlorhexidine gluconate (CHX), an antiseptic, has long been used as a first choice to manage plaque chemically, and will be the main comparison used throughout this review. However, its use has led to adverse effects such as mucosal irritation, tooth staining, and altered taste [Cardoso Nicolini et al. 2021 (1)]. Conversely, ozone offers an alternative with minimal side effects and similar broad-spectrum antimicrobial activity (2). Additionally, ozone does not produce antimicrobial resistance unlike antibiotics and some chemical antiseptics (3).

**Figure 1:**
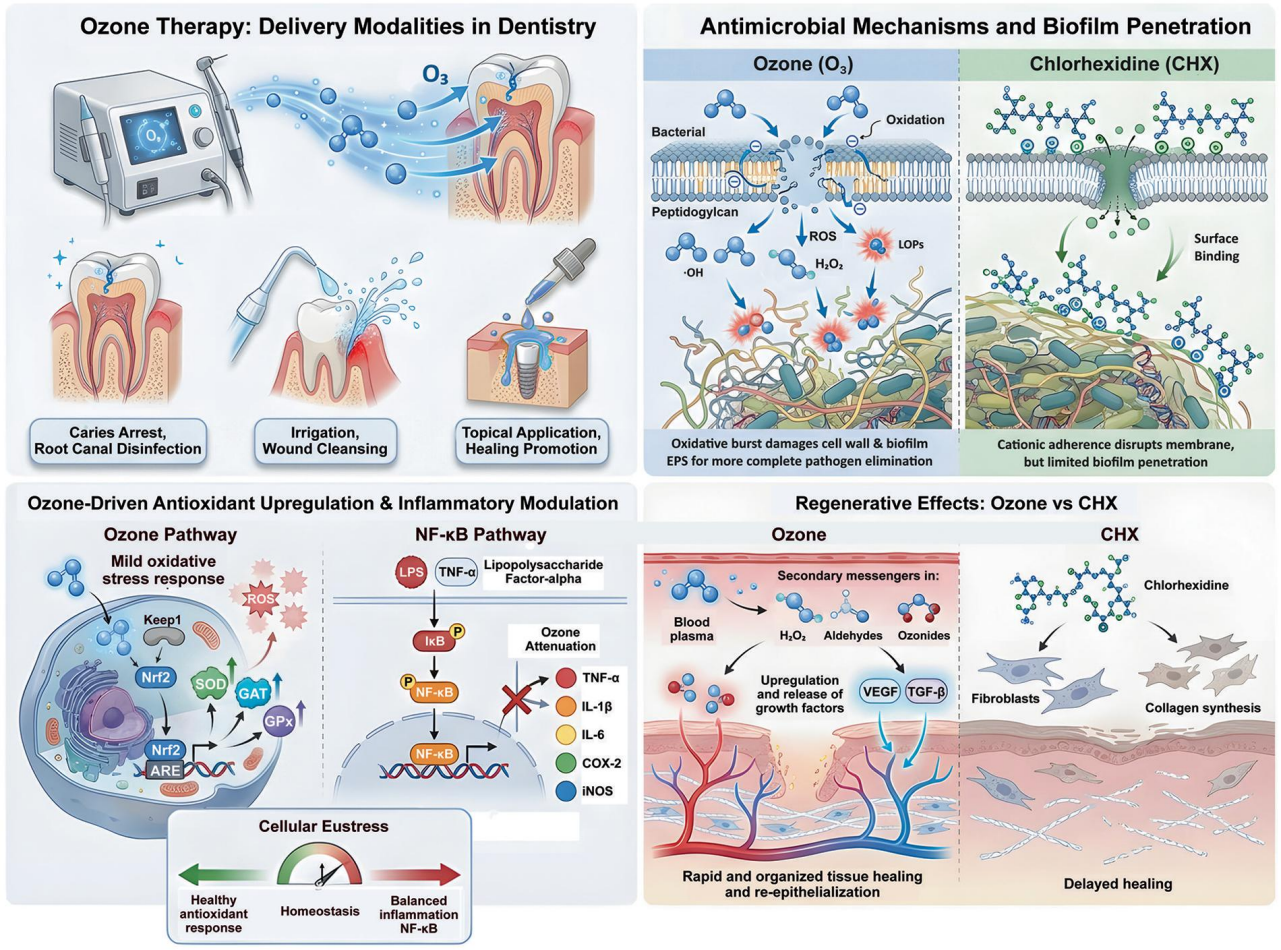
Comprehensive mechanistic comparison of ozone therapy vs. chlorhexidine in dentistry. (Top Left) Ozone therapy delivery modalities include gaseous ozone for caries arrest and root canal disinfection, ozonated water for irrigation and wound cleansing, and ozonated oils for topical application and healing promotion. (Top Right) Antimicrobial mechanisms and biofilm penetration. Ozone (O₃) generates reactive oxygen species (ROS) including hydroxyl radicals (·OH), hydrogen peroxide (H₂O₂), and lipid oxidation products (LOPs) that oxidatively damage bacterial cell walls and penetrate biofilm extracellular polymeric substance (EPS) for complete pathogen elimination. In contrast, chlorhexidine (CHX) achieves only surface binding through cationic adherence, with limited biofilm penetration due to EPS barrier. (Bottom Left) Ozone-driven antioxidant upregulation and inflammatory modulation. Left: Ozone pathway showing mild oxidative stress triggering Nrf2 release from Keap1, nuclear translocation, binding to antioxidant response elements (ARE), and upregulation of protective enzymes (SOD, CAT, GPx). Right: NF-*κ*B pathway showing ozone attenuation of inflammatory signaling by reducing bacterial triggers (LPS, TNF-α) and suppressing pro-inflammatory mediator production (IL-1β, IL-6, COX-2, iNOS). Center: Cellular eustress gauge illustrating homeostatic balance between healthy antioxidant response and controlled inflammation. (Bottom Right) Regenerative effects. Ozone generates secondary messengers (H₂O₂, aldehydes, ozonides) in blood plasma that upregulate growth factors (VEGF, TGF-*β*), promoting rapid angiogenesis, organized tissue healing, and re-epithelialization. CHX impairs fibroblast viability and collagen synthesis, resulting in delayed healing. ARE, antioxidant response elements; CAT, catalase; CHX, chlorhexidine; COX-2, cyclooxygenase-2; EPS, extracellular polymeric substance; GPx, glutathione peroxidase; IL-1β, interleukin-1 beta; IL-6, interleukin-6; iNOS, inducible nitric oxide synthase; I*κ*B, inhibitor of kappa B; LPS, lipopolysaccharide; LOPs, lipid oxidation products; Nrf2, nuclear factor erythroid 2-related factor 2; NF-*κ*B, nuclear factor kappa B; ROS, reactive oxygen species; SOD, superoxide dismutase; TGF-*β*, transforming growth factor-beta; TNF-α, tumor necrosis factor-alpha; VEGF, vascular endothelial growth factor.

Ozone therapy in dentistry is administered primarily through three different forms such as gaseous ozone, ozonated water, and ozonated oils (see Figure 1 – top left panel). Each of these different modalities have their own advantages and disadvantages depending on the patient and procedure. Gaseous ozone is administered chairside using specialized devices such as HealOzone (KaVo) or Prozone, which allows for a steady and controlled delivery of ozone gas directly to the patient's teeth and tissues. This form of administering ozone therapy is primarily useful in managing dentin hypersensitivity, disinfection of carious lesions, and reducing/eliminating microbial biofilms (4, 5). Ozonated water can be easily administered chairside through standard dental irrigation systems. The antimicrobial effects identified in ozonated water have shown to combat common pathogens found in periodontal disease and root canal infection, while also displaying less cytotoxic effects than common agents such as sodium hypochlorite (6, 7). Ozonated oils on the other hand combine the oxidative power of ozone with the stability of vegetable oils. The combination of these properties makes ozonated oils particularly advantageous in treating mucosal lesions, extraction sites, and implant sites due to their wound healing and anti-inflammatory properties (8, 9). Together, these delivery forms demonstrate the versatility of ozone therapy as a minimally invasive adjunct treatment in modern dentistry.

Ozone has been administered for therapeutic purpose using varying dosages (1 μg/mL up to 100 μg/mL) to treat osteoarthritis, tissue healing, and lower back pain (Serra et al. 2022). Of the different forms, ozonated water has sparked the most attention in dentistry due to its quick application and ability to manage microbial infections in the oral cavity. Ozone's half-life in distilled water is approximately 30 min. After 30 min, ozone (O₃) decomposes into diatomic O_2_ and an active singlet O_2_ molecule which has a high redox value, making ozone a strong disinfectant with stronger ability to degenerate and strip electrons from microbes [Khaimov and Tobias, 2020 (10)]. With a high redox value (2.07 V) and broad therapeutic index, ozone has the ability to combat a range of oral pathogens including bacteria, viruses, fungi, and biofilm (11, 12).

### Search strategy/study selection

A comprehensive literature search was conducted utilizing PubMed & Goggle Scholar to identify clinically relevant studies examining the benefits of ozone therapy in dentistry. Search terms included combinations of the following terms: “ozone therapy”, “ozonated water”, “ozonated oil”, “periodontics”, “endodontics”, “oral surgery”, “prosthodontics”, “orthodontics”, “restorative dentistry”, and “dentistry”. The initial search resulted in 44,852 records. Articles were screened and selected based on their relevance to the topic of this narrative review, study design, and applicability to clinical practice. Preference was given to randomized controlled trials, clinical studies, systematic reviews, and recent peer-reviewed publications. Studies that were unrelated to dentistry or ozone therapy, and articles that lacked evidence/credibility were excluded.

**Figure 2:**
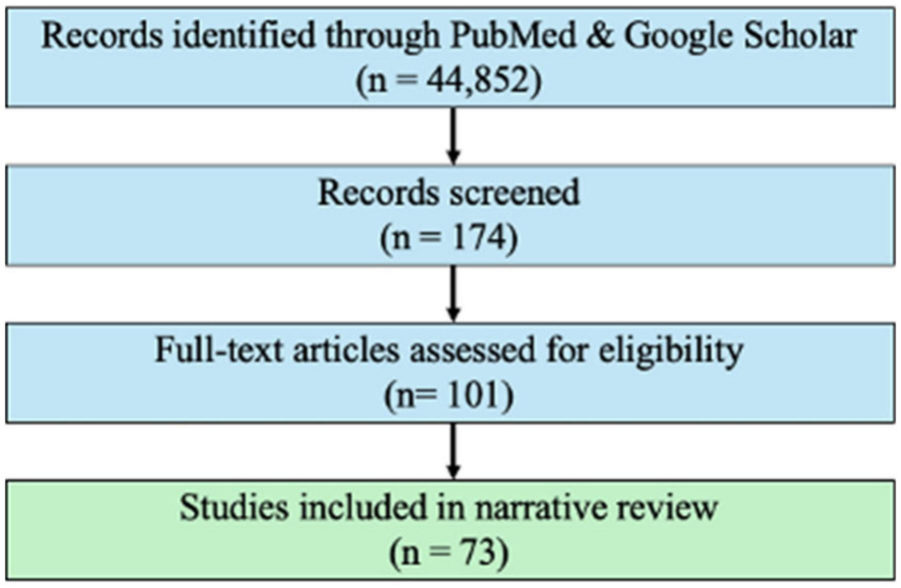
Literature search and study selection.

Following the screening process, 174 articles were selected for further evaluation. Assessments on full-text articles were done so for eligibility on 101 articles based on criteria, resulting in 73 studies that met the criteriafor this narrative review. Full-text articles were further evaluated based on their results and relevance to the topic. Although the manuscript is presented as a narrative review, elements of the PRISMA framework were implemented to improve transparency in study identification and screening. The summary for study selection can be summarized in Figure 2.

### Mechanism of action and biological properties in contrast to CHX

Ozone is mediated through complex host pathways which give rise to its antimicrobial, anti-inflammatory, and regenerative properties. In this section of the paper, the multiple mechanisms through which ozonated gas or water exhibits its vital properties will be explored. In addition, its comparison to chlorhexidine gluconate (CHX), the most widely used antiseptic in dentistry, is reviewed (see Figure 1, Table 1).

**Table 1:** Ozone therapy modalities.

Form of ozone	Mechanism of action	Main uses in dentistry
Gaseous ozone	Oxidative burst that disrupts microbial cell walls and inactivates pathogens	Caries management Root canal disinfection Hypersensitivity relief
Ozonated water	Penetrates tissues and biofilms through the release of reactive oxygen species	Periodontal irrigation Surgical site disinfection Root canal disinfection
Ozonated oils	Ozone reacts with unsaturated fatty acids (vegetable oils) and forms ozonides that slowly release oxygen	Topical application Dry socket Mucosal lesions

### Antimicrobial effects

Ozone's antimicrobial effect is primarily driven by the fact that it is a powerful oxidizing agent. Ozone exhibits a redox potential (+2.07 V) which is higher than that of hydrogen peroxide or hypochlorous acid (13, 14). This is important as a high redox value means higher efficiency at removing electrons from microbial cell walls. A high redox potential enables ozone to kill planktonic bacteria and disrupt resistant biofilms. These biofilms are often difficult for weaker antisepctics to penetrate. Ozone does so by rapidly decomposing into reactive oxygen species such as hydrogen peroxide, hydroxyl radicals, and lipid oxidation products (LOPs) when let into aqueous environments. These species cause oxidative damage to the microbe's cell walls and cytoplasmic membranes, resulting in rapid cell death (7). Clinically, this means that ozone shows faster microbial killing and broader antimicrobial spectrum due to its higher redox value than other antiseptics (15).

CHX functions differently in terms of its antimicrobial activity since it uses a cationic bisbiguanide mechanism. Bacterial cell walls are negatively charged, therefore the CHX molecules which are positively charged are attracted to the cell walls and tightly bind to them (16). This bind explains the cationic bisbiguanide mechanism. Once bound, CHX disrupts the integrity of the bacterial membrane, increasing permeability and causing leakage of intracellular components (17). At low concentrations (0.02%–0.06%), CHX acts as a bacteriostatic agent by inhibiting bacterial growth rather than directly killing cells. This happens when osmotic equilibrium is distributed, preventing normal nutrient transport and cell division. At higher concentrations (0.12%–0.2%), CHX becomes bactericidal, leading to precipitation of cytoplasmic proteins and collapse of cellular integrity. This causes bacterial death (16, 17).

Although CHX is effective against a wide range of bacteria, fungi, and some viruses, a major limitation is its lack of biofilm-penetrating activity. Biofilms consist of microbial communities encased in an extracellular polymeric substance (EPS) composed of polysaccharides, proteins, and nucleic acids. The EPS acts as a physical and chemical barrier that traps CHX molecules before they can diffuse to the deeper layers of the biofilm (18, 19). As a result, CHX primarily eliminates microorganisms at the biofilm surface, while bacteria in the protected core can survive and recolonize once treatment ceases (20). Ozone, by contrast, produces small, highly reactive oxidants that degrade the EPS matrix itself, enabling deeper penetration and more complete microbial elimination (15).

### Anti-inflammatory and immunomodulatory effects

1. Ozone's anti-inflammatory effects are primarily accomplished by the modulation of the Nrf2 antioxidant pathway and suppression of the NF-*κ*B inflammatory pathway (see Figure 1).
  a. Ozone and the Nrf2 Pathway

In host tissues, under homeostatic conditions, the transcription factor nuclear factor erythroid 2–related factor 2 (Nrf2) is kept bound in the cytoplasm by its repressor protein Keap1. When ozone is introduced, it produces a mild oxidative stimulus that disrupts the Nrf2-Keap1 interaction thus allowing Nrf2 to translocate to the nucleus. Once in the nucleus, Nrf2 binds to antioxidant response elements (AREs). This interaction upregulates antioxidant and cytoprotective enzymes such as superoxide dismutase (SOD), catalase (CAT), and glutathione peroxidase (GPx) (3, 21). These enzymes act in a coordinated manner to neutralize reactive oxygen species (ROS) and maintain redox balance. Superoxide dismutase (SOD) serves as the first line of defense by converting superoxide radicals (O₂•–) into hydrogen peroxide (H₂O₂) and molecular oxygen (O₂) (22, 23). Hydrogen peroxide, while less reactive than superoxide, can still cause oxidative injury if it accumulates; therefore, catalase (CAT) rapidly converts H₂O₂ into water and oxygen, eliminating bulk quantities of the molecule (24). At the same time, glutathione peroxidase (GPx) provides additional protection in the cytoplasm and mitochondria by using glutathione to reduce both hydrogen peroxide and lipid peroxides, thereby safeguarding cell membranes from oxidative damage (25).

b. Ozone and the NF-*κ*B Pathway

In contrast to Nrf2, the nuclear factor kappa B (NF-*κ*B) pathway promotes inflammatory responses. NF-*κ*B is usually kept in the cytoplasm by its inhibitor protein, I*κ*B. I*κ*B is phosphorylated by the I*κ*B kinase (IKK) complex and degraded when microbial products such as lipopolysaccharides (LPS) or host-derived signals such as tumor necrosis factor-alpha (TNF-α) encounter it. When I*κ*B is degraded, NF-*κ*B is released and moves to the nucleus of the host. When in the nucleus, NF-*κ*B drives the making of pro-inflammatory mediators, including TNF-α, interleukin-1 beta (IL-1β), interleukin-6 (IL-6), cyclooxygenase-2 (COX-2), and inducible nitric oxide synthase (iNOS) (26). Thus, causing pain and swelling.

Ozone therapy has been shown to attenuate NF-*κ*B activation through multiple mechanisms. First, by reducing microbial load, ozone limits the availability of bacterial products such as LPS that would otherwise trigger NF-*κ*B activation (5). Second, ozone shifts the intracellular redox state toward an antioxidant-dominant environment via Nrf2-mediated upregulation of superoxide dismutase, catalase, and glutathione peroxidase, thereby dampening redox-sensitive NF-*κ*B signaling (3, 21). Third, experimental evidence suggests that ozone may stabilize I*κ*B, preventing its degradation and keeping NF-*κ*B sequestered in the cytoplasm (21). Finally, ozone directly decreases transcription of downstream mediators, including TNF-α, IL-1β, IL-6, COX-2, and iNOS, thereby reducing prostaglandin and nitric oxide production that contribute to pain and inflammation (11, 12). To summarize, in the early phases of wound healing, nitric oxide (NO) exerts an anti-bacterial role in wound debridement, then later on induces an angiogenic effect, collagen synthesis and wound maturation. In addition, nitric oxide in controlled concentrations help reduce pain associated with chronic inflammation by stimulating anti-inflammatory and antinociceptive responses in the tissue (27).

### Tissue regeneration and angiogenesis

Ozone also demonstrates keen tissue regeneration properties (See Figure 1 – bottom right panel). When ozone interacts with plasma and other biological fluids, it creates reactive oxygen species and lipid oxidation products which serve as secondary messengers that activate cell signaling pathways directly stimulating tissue healing and remodeling. Some of these secondary messengers include hydrogen peroxide, aldehydes, and ozonides (2, 3). Markedly, ozone has the ability to promote angiogenesis, creating a favorable environment for tissue repair through the nutrient absorption and oxygen delivery achieved from increased vascularization. Ozone does so by upregulating vascular endothelial growth factor, otherwise commonly known as VEGF, transforming growth factor-beta (TGF-*β*), and pro-angiogenic signals fibroblast growth factor 2 (FGF2) and platelet -derived growth factor (PDGF) (12). Conversely, CHX has been shown to impair fibroblast viability and collagen metabolism, delaying tissue regeneration and repair (28).

### Clinical delivery of ozone in dentistry

Ozone can be delivered in various forms such as gas, ozonated water, and ozonated oils—each with their own unique clinical applications across various dental specialties such as periodontics, endodontics, restorative dentistry, and oral surgery (29). Each of these modalities have shown their therapeutic potential in oral medicine as adjunctive treatments, particularly due to the antimicrobial and anti-inflammatory properties of ozone therapy. This review will now elaborate on the different uses of ozone therapy and their uses in the subfields of dentistry.

### Gaseous ozone

Ozone gas is produced through the conversion of oxygen into ozone through either a corona discharge or UV light method inside a dental ozone generator. Delivery of gaseous ozone is done so through a process that involves sealing the desired area of effect (e.g., carious lesion, exposed dentin, etc.) with a silicone cup or handpiece to administer the gas. Currently, the two primary dental ozone delivery systems that are commercially available and approved for clinical use in the United States are HealOzone (KaVo). and Prozone. HealOzone administers ozone gas at approximately 2,100 ppm and can do so through a sealed handpiece. This process involves sealing the area of effect (e.g., a single tooth) with a silicone cup and applying gaseous ozone for the recommended time limit, followed by removal of any residual ozone for patient safety. HealOzone is advantageous in managing hypersensitivity, carious lesions, and general microbial/plaque reduction on the surfaces of teeth. Unlike HealOzone, Prozone does not utilize a sealed delivery system like its counterpart and delivers ozone in a more open application method. Although there are other devices used in other countries such as Ozonytron and OzoneDTA, they are not readily available in the US due to a lack of standardization, regulatory approval, and clinical testing, making HealOzone and Prozone the only two widely accessible systems in the United States. Studies have demonstrated that gaseous ozone delivered via HealOzone (KaVo) and Prozone can significantly reduce microbial load and contribute to decreasing sensitivity of exposed dentin (4, 5). Furthermore, laboratory investigations suggest that gaseous ozone may reduce dentinal hypersensitivity by enhancing dentinal tubule permeability and facilitating deeper penetration and uptake of remineralizing and desensitizing agents, including calcium, phosphate, fluoride, and resin-based desensitizers (30). The primary therapeutic effect of HealOzone is attributed to the oxidative properties of ozone, which promotes the penetration of dentinal tubules and disrupts bacterial biofilms while promoting mucosal health (5). Although Prozone does not utilize a sealed delivery system, it displayed similar results in the alleviation of dentinal hypersensitivity (4). A primary advantage of these devices is the minimally invasive treatment that can be offered chairside to treat infected tissue.

Although clinical findings of gaseous ozone treatment are optimistic, there is some conflicting evidence from randomized controlled trials studying hypersensitivity. A randomized triple-blind trial suggested that HealOzone presents a significant reduction in pain over the course of eight weeks, however the results were not significantly different to what was found in the placebo control (4, 31). Similarly, a study utilizing Prozone reported no significant difference in pain reduction compared to placebo, underscoring the need for further research to clarify the clinical efficacy of ozone therapy (32). In contrast, a more recent randomized controlled clinical trial by D’Amario et al. (33) comparing ozone gas therapy with diode laser treatment for dentin hypersensitivity demonstrated a statistically significant reduction in hypersensitivity at approximately six months in the ozone group (*p* = 0.00026), suggesting a superior therapeutic effect of ozone under these conditions.

### Ozonated water

Ozonated water has displayed potential in the literature as a powerful irrigation solution in both endodontic and periodontal cases. Administration of ozonated water may be done utilizing standard dental irrigation systems which allows for a convenient adjunctive method to eliminate bacterial agents in periodontal and endodontic procedures (6). Pathogens found in root canal infections such as *Enterococcus faecalis* have been neutralized by ozonated water therapy, highlighting its strong antimicrobial activity. Ozonated water therapy produced a 98% reduction in bacterial load, comparable to sodium hypochlorite (NaOCl) and chlorhexidine (CHX) *in vitro* (34). Furthermore, ozonated water significantly reduced *E. faecalis* and *S. mutans* levels and provided a less cytotoxic alternative treatment to cultured oral epithelial cells (7, 10). In periodontal therapy, ozonated water can reduce plaque accumulation, bleeding on probing, and inflammation. A systematic review found that adjunctive treatment with ozonated water irrigation in periodontitis patients produced significantly improved clinical parameters such as probing depths and plaque index (35). A key advantage of ozonated water therapy is its low cytotoxicity and regenerative potential. Furthermore, administration of low concentrations of ozonated water has suggested to promote dental pulp proliferation, suggesting that ozonated water may be a favorable agent in dental pulp therapy and regenerative endodontics (10).

### Ozonated oils

Ozonated oils, most notably olive oil, have emerged as a valuable adjunctive treatment in treating dental issues (e.g., mucosal lesions, extraction sites, and peri-implant tissues) due to their ability to entrap and release oxygen and ozone (8, 9). Ozonated olive oil is a common agent in studies using ozonated oils. This is simply olive oil that has been infused with ozonated gas to create a thick ointment type agent that presents with wound healing and antimicrobial properties. Ozonated olive oil has been examined due to its potential in reducing postoperative pain and promotion of wound healing. In surgical extraction cases patients treated with ozonated olive oil showed comparable pain relief to conventional analgesics, including ibuprofen and acetaminophen, highlighting its potential in providing safe and alternative treatment of postoperative pain (36). Furthermore, there was no significant difference in plaque index and gingival index between patients treated with ozonated olive oil or chlorhexidine gel when managing peri-implant mucositis, suggesting that ozonated olive oil may prove to be a non-cytotoxic alternative for implant care (37). Evidence presented from systematic reviews helps support the clinical application of ozonated oils in oral medicine. Ozonated oils were found to be effective across a wide spectrum of oral mucosal lesions, such as gingivitis, ulcers, stomatitis, alveolitis, and herpetic lesions, while exhibiting no adverse effects, underlining their potential as a safe and versatile therapeutic option (38). Furthermore, ozonated oil treatment results were comparable to those of common healing agents such as alvogyl, chlorhexidine, and other common antiseptics used in wound healing. Another encouraging area of research is the use of ozonated oils with implant extraction sites. For example, a recent study found that when applying ozonated olive oil to extraction sites, there was significant enhancement of osseointegration, soft tissue regeneration, and a 100% implant survival rate after follow-up compared to conventional postoperative management (39).

### Applications across dental specialties

Research in clinical applications of ozone therapy has been steadily documented over the years in various fields of dentistry such as restorative dentistry, endodontics, periodontics, pediatric dentistry, and oral surgery (30, 78). The powerful antimicrobial and anti-inflammatory properties demonstrated allow for this therapeutic model to be a versatile way to provide adjunctive treatment, resulting in improved outcomes, in various subfields of dentistry. This section will focus on the uses of ozone within the different specialties.

### Restorative dentistry

Ozone therapy has been associated with prevalent antimicrobial effects against cariogenic bacteria and is beneficial in managing non-cavitated lesions. Early clinical data focused primarily on caries arrest, with reports of 100% caries arrest after just 18 months of ozone therapy (Holmes et al. 2003). Ozone treated lesions were reported to show significantly higher caries arrest rates at each of the evaluation intervals (69% vs. 4% at 3 months and 92% vs. 24% at 6 months) when compared to the control group (conventional remineralization management with no ozone application). Findings are consistent with previous studies in the field further supporting the role of ozone therapy in early caries arrest and management (40, 41). In more recent studies, the shift in ozone therapy used in restorative dentistry has been towards its use as a cavity disinfectant before restoration, mostly due to the ability in preserving tooth structure in cavity preparations.

Populations of the well-known key contributors to carious lesion formation such as *Streptococcus mutans* and *Lactobacillus casei*, can be significantly reduced just after 30 s of ozone exposure (42). A randomized clinical trial evaluated ozone therapy with and without an adjunctive remineralization solution and found that ozone therapy produced comparable remineralization of non-cavitated pit and fissure caries (ICDAS 1-2) in children (Atabek 2011). Children received ozone therapy for 40 s, and over a six-month period, a significant improvement in their non-cavitated lesions were noted (*p* < 0.001). However, these findings were only applicable to smaller cavitated lesions and did not include larger cavitated lesions.

### Endodontics

Endodontics cases present the primary challenge of achieving thorough disinfection of complex root canal systems to eliminate residual pathogens such as *Enterococcus faecalis*. Sodium hypochlorite (1%–5.25%) remains the standard of care for root canal disinfection (43–45); however, multiple studies have reported potential cytotoxic effects associated with its use (46–49). Early investigations demonstrated that sodium hypochlorite even at low concentrations of 0.01% can cause complete hemolysis of red blood, while higher concentrations may induce severe soft tissue damage (50). Several studies have documented the antimicrobial efficacy of ozonated water against endodontic pathogens (75). Ozonated water (24 mg/L) exhibited antimicrobial activity comparable to sodium hypochlorite (2.5%) in eliminating keystone pathogens such as *E. faecalis* (51). Similarly, Savitri et al. (52) evaluated the antimicrobial efficacy of ozonated water at a working concentration of 4 mg/L, chlorhexidine (2%), and sodium hypochlorite (5.25%) using agar diffusion and direct contact tests against five common endodontic pathogens. When tested in identical conditions against five different endodontic pathogens (*E. faecalis*, *S. mutans*, *S. aureus*, *C. albicans*, *K. rhizophila*), 2% chlorhexidine demonstrated the greatest antimicrobial effect, followed by 5.25% sodium hypochlorite, and ozonated water (4 mg/L) exhibited a smaller antimicrobial effect compared to both chlorhexidine and sodium hypochlorite. The inhibition zones across the five endodontic pathogens varied with chlorhexidine inhibition zones going up to as high as 25 mm, sodium hypochlorite demonstrated inhibition zones from 9 to 14 mm, while ozonated water had smaller inhibition zones across the board from 8 to 12 mm under identical conditions. Additional studies have explored the potential benefits of ozonated water as an alternative to more cytotoxic irrigants such as sodium hypochlorite in endodontic therapy (77). Working concentrations of 2, 4, 8, and 16 mg/L of ozonated water were evaluated to determine cytotoxic thresholds Küçük et al. (10). Dental pulp cells exposed to lower concentrations (2 and 4 mg/L) for 48 h demonstrated improved viability, whereas higher concentrations (8 and 16 mg/L) were associated with cytotoxic effects. In contrast, sodium hypochlorite at commonly used working concentrations of 1% and 3% has been shown to exhibit cytotoxicity depending on the length of exposure (46, 53). Notably, Mukundan et al. (53) reported that 3% sodium hypochlorite resulted in approximately 60% mortality in brine shrimp populations after 24 h. Overall, these findings suggest that ozonated water at lower concentrations (2–4 mg/L) may represent a viable alternative irrigant to higher-concentrated sodium hypochlorite solutions in situations where cytotoxicity is a clinical concern.

### Periodontics/oral surgery

Periodontics represents one of the most well documented subfields in studying application and benefits of ozone therapy in dentistry. Chronic periodontitis is a prolonged inflammatory condition in which supporting tooth structure is destroyed due to the accumulation of pathogenic biofilm and host immune responses. Although scaling and root planing (SRP) is the gold standard in periodontal therapy, there are other adjunct treatments that can be used to enhance clinical outcomes. Ozonated water has been widely studied as a potential adjunctive treatment to SRP. Multiple studies have shown the use of ozonated water in addition with SRP results in significant improvements in post operative healing. Patients have been noted to show significant reductions in probing depth, bleeding on probing, and plaque index when treated with ozonated water compared to chlorhexidine (1, 54). Interestingly, subgingival irrigation in conjunction with ozonated water following SRP resulted in statistically significant reductions in probing pocket depths over a two-month period compared to distilled water irrigation. Subgingival irrigation with ozonated water following SRP produced a statistically significant reduction in probing pocket depths (55). Systematic review data further support these findings. Periodontal parameters were evaluated before and after treatment, including PD, GI, BOP, and PI, and clinical attachment loss (CAL). This data showed that ozone therapy combined with SRP produced the most statistically significant improvements in PD and GI compared to SRP alone (*p* < 0.05) (54). Ozonated oils have also been evaluated as adjunct treatments to SRP. Randomized clinical trial data and systematic review findings consistently demonstrated that ozonated olive oil irrigation used alongside SRP produced statistically significant reductions in probing pocket depth and plaque index within one month of treatment in patients with chronic periodontitis (56, 57). To further support these findings, topical ozone treatment in conjunction with SRP resulted in mean improvements of 0.32 mm in CAL and 0.41 mm in PD, suggesting a positive overall clinical benefit (58). Other than non-surgical periodontal therapy, ozonated gas has revealed favorable results in post-extraction socket and surgical healing. Notably, improved postoperative healing and reduced infections have been recorded when surgical sites have been treated with ozone gas (5, 59). Additional clinical studies have reported reduction in postoperative inflammation, swelling, and pain following ozone gas therapy in third molar extractions (29, 60, 61). Patients receiving adjunctive ozone treatment demonstrated reduced analgesic consumption, improved quality of life, and decreased postoperative pain during the first five days following surgery. Beyond traditional methods in periodontal therapy, recent literature has highlighted the importance of maintaining favorable conditions for healing during implant and regenerative surgical procedures. Guided bone regeneration (GBR) procedures heavily focus on factors such as infection control, wound healing, and surgical stability to promote per-implant health and treatment success (62). Due to its antimicrobial and anti-inflammatory properties, ozone therapy may serve as a beneficial adjunctive treatment in these procedures by promoting improved postoperative healing and reducing inflammation. A study investigating the inhibitory effect of gaseous ozone on 23 microbial strains, 17 of which were periodontal pathogens, concluded that ozone had bactericidal effect on *Porphyromonas gingivalis*, *Fusobacterium nucleatum, Aggregatibacter actinomycetemcomitans* and anaerobic gram negative and gram positive bacteria after 18 and 24 s of exposure (63). A recent systematic review examined ozone therapy in periodontal and peri-implant surgical wound healing and reported improvements in postoperative healing and reductions in inflammatory parameters across various clinical applications (64). Even though additional long-term clinical studies and standardized ozone protocols are still needed, current findings suggest ozone therapy may have potential benefits in regenerative periodontal and peri-implant surgical therapy.

### Prosthodontics and orthodontics

Maintaining effective plaque control around fixed appliances remains a persistent challenge in both prosthodontics and orthodontics. The presence of fixed prostheses and orthodontic appliances creates additional areas for plaque accumulation and retention. As a result, adjunctive strategies aimed at improving plaque control and reducing inflammation have gained increased attention. Recent studies have highlighted the uses of ozonated water as an adjunctive tool for management of plaque and inflammation in prosthodontics and orthodontics. When comparing ozonated water and chlorhexidine as treatments for plaque reduction, ozonated water was found to be far more effective in reducing plaque index, and bleeding on probing scores in orthodontic patients, therefore improving periodontal health and reducing prevalence of caries (Cosola et al. 2019). Ozonated water was significantly more effective in reducing plaque index (68.7% to 24.3%) and bleeding scores (32.8% to 4.70%), suggesting that ozone therapy may be a more effective and patient-friendly alternative treatment. Furthermore, in pediatric patients undergoing orthodontic treatment where there are an elevated caries risk and oral hygiene is often difficult to maintain, ozonated water treatment significantly reduced *Streptococcus mutans* levels in patients (65)). One study assessed subgingival irrigation utilizing ozonated water and found that there was a significant reduction in gingival inflammation compared to saline irrigation, reinforcing ozone therapy's anti-inflammatory and antimicrobial properties (66). In orthodontic patients with fixed appliances, ozonated water irrigation was associated with reductions in plaque levels (71.4%), gingival inflammation (74.6%), gingival bleeding (93%), and probing pocket depths (*p* < 0.01) when compared to conventional protocols (67). These findings were consistent with earlier work demonstrating significant improvements in plaque accumulation and gingival inflammation using ozonated water at a concentration of 0.01 mg/L (68). More recently, orthodontic patients with fixed appliances experiencing gingival inflammation were treated using ozonated water (group 1) and low-level diode laser therapy (group 2) (69). Although laser treatment had a marginal increase in efficacy, ozonated water still was able to produce a comparable clinical response (*p* < 0.05), supporting the practicality for routine clinical use in orthodontic patients with fixed appliances.

### Limitations

Despite the supporting data of the benefits and uses of ozone therapy across the different specialties of dentistry, there are several limitations. One of the primary concerns with ozone therapy is the potential for interference with the adhesion of restorative materials. Studies have demonstrated that ozone gas can hinder the bond strength of composite to enamel/dentin, which can cause these restorations to fail (29, 70). Another issue with the expanded use of ozone therapy is the lack of clear and concise protocols for study design. Without standards set concerning dosage, duration, and delivery, it makes it extremely difficult to compare studies to one another and for clinicians to properly implement its use into their practices (2, 71). The consistent variability in results makes it hard to reproduce findings and allow for effective clinical decision-making. For example, systematic reviews by Thome et al. (58) and Liu et al. (54) both had similar positive results in the reduction of probing pocket depths and bleeding on probing, but the significance of their findings vary because the studies examined have varying ozone concentrations, duration of treatment, and follow-up time. This makes it extremely difficult to compare results between studies because there is not a unifying parameter to examine between studies. Continuous research is needed to better understand ozone therapy efficacy in oral medicine and to standardize its treatment to allow for easier implementation into clinical practice.

Furthermore, ozone therapy systems can present a financial barrier to some clinicians, with device costs typically ranging from approximately $2,000 to $5,000 depending on the system and manufacturer. In fact, professional-grade systems may cost clinicians up to $5,000, which, seems modest compared to costs of other dental equipment, but may still limit adoption depending on varying factors such as practice revenue and patient population. Although there is a lot of literature available, there is still a shortage of randomized controlled trials with larger sample sizes. More RCT's and funding would be crucial in understanding how to better implement ozone therapy in clinical practice and its efficacy (72, 73). Although ozone therapy has resulted in significant antimicrobial activity in periodontal and endodontic cases, conflicting findings still exist regarding whether its antimicrobial efficacy is comparable or superior to conventional irrigants such as sodium hypochlorite and chlorhexidine (15, 71, 76). Similarly, although ozone therapy has shown favorable results in the management of dentin hypersensitivity, several clinical studies reported outcomes comparable to placebo or conventional treatment modalities rather than statistically significant improvements (4, 32). These inconsistencies make it difficult to determine if ozone therapy should be considered a replacement for conventional treatment methods or mainly used as an adjunctive therapy. Increasing exposure time to NaOCl was found to produce better microbial reduction than ozone therapy (15). There are noteworthy uses of ozone therapy in restorative dentistry; however, one clinical trial examining the effectiveness of sealants, ozone therapy, and fluoride varnish in premolars over the course of a year did not find statistically significant results in favor of ozone therapy (74). Additionally, the reported benefits of ozone therapy in combination with fluoride and sealants appear to be largely limited to specific patient populations, most notably pediatric patients with small, non-cavitated lesions (ICDAS 1-2) and increased dental anxiety (65). Although there is potential in ozone therapy application in prosthodontics and orthodontics, there is limited clinical data to evaluate. More research is needed to better understand the efficacy of ozone therapy and to establish standardized protocols (2, 71, 72).

A significant regulatory limitation in interpreting the evidence for ozone therapy is that the U.S. Food and Drug Administration (FDA) has not approved ozone therapy for any medical or therapeutic indication. In fact, according to 21 CFR § 801.415, ozone is explicitly characterized as “a toxic gas with no known useful medical application in specific, adjunctive, or preventive therapy.” (U.S. Food and Drug Administration 2025). Furthermore, devices purporting to deliver medical ozone that make health-related claims are subject to the Federal Food, Drug, and Cosmetic Act, and those without adequate supporting clinical data may be considered misbranded or adulterated (FDA, CPG Sec. 355.300). Indeed, the FDA has issued warning letters to companies marketing ozone-based devices for CPAP cleaning or disinfection that lack regulatory clearance, emphasizing that no ozone-based device is currently authorized for those medical claims (FDA 2024). While dental ozone delivery systems such as HealOzone and Prozone are used clinically in dentistry, they are not broadly FDA-approved for systemic or generalized medical disinfection claims. It is important to highlight that the absence of FDA approval does not necessarily prevent the use of ozone as an adjunctive tool within dentistry. In fact, many ozone-based applications discussed throughout the literature are utilized as adjunctive or alternative therapies that are intended to be used with conventional dental treatment rather than replacing the standard of care. Because of this regulatory status, much of the published literature exists in the context of alternative or adjunctive use, with variability in device standards, dosing protocols, and reporting transparency. Thus, the absence of comprehensive FDA approval underscores the need for rigorous, standardized clinical trials before ozone therapy can be recommended confidently in practice.

## Conclusion

Ozone therapy has exhibited its potential in several fields of dentistry through various modalities such as liquid, oils, and gas; making it extremely versatile in its delivery. Ozone therapy is often favorably compared to other agents in the literature such as sodium hypochlorite and chlorhexidine due to its significant anti-inflammatory, antimicrobial, and wound healing properties. Unlike these gold standard agents, ozone therapy does not present significant cytotoxicity problems. Current evidence appears strongest for the adjunct use of ozone therapy in periodontal therapy, postoperative wound healing, and management of early non-cavitated carious lesions. However, evidence regarding dentin hypersensitivity management, endodontic disinfection efficacy, and broader clinical implementation remains inconsistent across studies. Ozone therapy is limited due to its lack of standardization in protocol. Expanding research and funding of ozone therapy will allow for better implementation into everyday clinical practice. Furthermore, this will present clinicians with an alternative non-invasive method to provide dental treatment.
